# Anticancer properties of bacterial cellulose membrane containing ethanolic extract of *Epilobium angustifolium L*

**DOI:** 10.3389/fbioe.2023.1133345

**Published:** 2023-02-20

**Authors:** Magdalena Perużyńska, Anna Nowak, Radosław Birger, Paula Ossowicz-Rupniewska, Maciej Konopacki, Rafał Rakoczy, Łukasz Kucharski, Karolina Wenelska, Adam Klimowicz, Marek Droździk, Mateusz Kurzawski

**Affiliations:** ^1^ Department of Experimental and Clinical Pharmacology, Pomeranian Medical University in Szczecin, Szczecin, Poland; ^2^ Department of Cosmetic and Pharmaceutical Chemistry, Pomeranian Medical University in Szczecin, Szczecin, Poland; ^3^ Department of Chemical Organic Technology and Polymeric Materials, Faculty of Chemical Technology and Engineering, West Pomeranian University of Technology in Szczecin, Szczecin, Poland; ^4^ Department of Chemical and Process Engineering, Faculty of Chemical Technology and Engineering, West Pomeranian University of Technology in Szczecin, Szczecin, Poland; ^5^ Department of Nanomaterials Physicochemistry, Faculty of Chemical Technology and Engineering, West Pomeranian University of Technology in Szczecin, Szczecin, Poland

**Keywords:** bacterial cellulose, Epilobium angustifolium, cytotoxicity, colon cancer, anticancer therapy

## Abstract

*Epilobium angustifolium L.* is a medicinal plant well known for its anti-inflammatory, antibacterial, antioxidant, and anticancer properties related to its high polyphenols content. In the present study, we evaluated the antiproliferative properties of ethanolic extract of *E. angustifolium* (EAE) against normal human fibroblasts (HDF) and selected cancer cell lines, including melanoma (A375), breast (MCF7), colon (HT-29), lung (A549) and liver (HepG2). Next, bacterial cellulose (BC) membranes were applied as a matrix for the controlled delivery of the plant extract (BC-EAE) and characterized by thermogravimetry (TG), infrared spectroscopy (FTIR), and scanning electron microscopy (SEM) images. In addition, EAE loading and kinetic release were defined. Finally, the anticancer activity of BC-EAE was evaluated against the HT-29 cell line, which presented the highest sensitivity to the tested plant extract (IC_50_ = 61.73 ± 6.42 µM). Our study confirmed the biocompatibility of empty BC and the dose and time-dependent cytotoxicity of the released EAE. The plant extract released from BC-2.5%EAE significantly reduced cell viability to 18.16% and 6.15% of the control values and increased number apoptotic/dead cells up to 37.53% and 66.90% after 48 and 72 h of treatment, respectively. In conclusion, our study has shown that BC membranes could be used as a carrier for the delivery of higher doses of anticancer compounds released in a sustained manner in the target tissue.

## 1 Introduction

Fireweed (*Epilobium angustifolium* (L.) Holub.) is a well-known medicinal plant that grows naturally in many locations in the world, mainly in North America, Asia, and Europe (Lin et al., 2022; Dreger et al., 2020) (Kalle et al., 2020). The plant has well-known anti-inflammatory (Ferrante et al., 2020) (Nowak et al., 2021c; Zagórska-Dziok et al., 2021), antibacterial, anticancer (Kia et al., 2021; Ostrovska et al., 2017; Kowalik et al., 2022) (Maruška et al., 2017) and antioxidant properties (Lasinskas et al., 2020) (Nowak et al., 2019; Karakaya et al., 2020). Traditionally, the leaves infusion of this plant is used as a remedy for headaches, colds, gastrointestinal and prostate disorders (Lasinskas et al., 2020). It is also applied topically as an antiseptic for wounds and various skin and mucosa diseases (Karakaya et al., 2020). The therapeutic properties of *E. angustifolium* are related to high content of valuable secondary metabolites, mainly polyphenols. Among the most active components are flavonoids, phenolic acids, and tannins (Nowak et al., 2021c; Nowak et al., 2022; Dacrema et al., 2020). However it should be pointed out that polyphenols possess a low oral bioavailability related to extensive biotransformation mediated by the liver, intestine, and gut microbiot (Luca et al., 2020; Xiao and Högger, 2015; Di Lorenzo et al., 2021). According to Clifford et al., less than 5% of the ingested polyphenols are absorbed and reach the plasma unchanged (Clifford, 2004). A promising strategy to overcome low bioavailability of phytochemicals is the application of delivery systems that could modulate the pharmacokinetics of active compounds (Aqil et al., 2013). Moreover, application of drug carriers allows sustained release of active compounds in the target tissue. Particularly, in the case of solid tumours, localized delivery of chemotherapeutic agents may improve therapy outcomes and decrease side effects compared to systemic chemotherapy (Seib et al., 2013; Chiu et al., 2014). Direct delivery of drugs to tumour tissues may also be an alternative for patients who either cannot undergo surgery in the case of surgery failure or contraindications of systemic chemotherapy. Another possible strategy might constitute application of drug carriers in neoadjuvant therapy (reduction of tumour volume before surgery) or as adjuvant therapy (prevention of tumour recurrence after surgery) e.g., in patients with stage III colon cancer (Taieb and Gallois, 2020). Local drug delivery is successfully used for several tumours, including bladder (Nilsson et al., 2001), glioma (Kleinberg, 2012), and prostate (Goldspiel and Kohler, 1991; Ren et al., 2022).

Biopolymers (including gelatin, chitosan, collagen, and cellulose) have been extensively investigated for their biomedical applications because of their eco-friendly synthesis, availability in large quantities, and first of all - biocompatibility. Cellulose is the most abundant biomaterial on Earth, synthesized by various organisms, including bacteria, plants, and even some animals (Klemm et al., 2006). However, bacterial cellulose (BC) has several advantages compared to the most popular plant-derived cellulose, such as considerably higher crystallinity (80%–90%), water absorption capacity, and degree of polymerization (up to 8,000) (Moniri et al., 2017). Moreover, BC is characterized by higher purity, as it does not contain hemicellulose, lignin, or natural pigments (Thomas et al., 2018). It also shows higher heat resistance, which enables sterilization by autoclaving (Bodin et al., 2007), which is especially important in biomedical applications. Furthermore, its high flexibility and porosity make it an excellent biomaterial that could be used as an implantable matrix for localized drug delivery (Abeer et al., 2014). Some studies support the use of cellulose membranes as carriers for drugs (Cacicedo et al., 2018) or plant extracts with potential anticancer properties (Taokaew et al., 2014; Taokaew et al., 2021; Subtaweesin et al., 2018).

Despite the wide use of *E. angustifolium* in natural medicine, there are no reports about its controlled delivery to target tissues. The current study is the first report describing the anticancer properties of BCs containing ethanolic extract of *E. angustifolium* (EAE). In the present study, we evaluated anticancer properties of *E. angustifolium* against selected (based on literature reports (Ostrovska et al., 2017; Kowalik et al., 2022; Maruška et al., 2017; Pei et al., 2019) cancer cell lines, including melanoma (A375), breast (MCF7), colon (HT-29), lung (A549), liver (HepG2) and primary human fibroblasts as a control. Next, the varied concentrations of the EAE were loaded into BC and characterized by means of thermogravimetry (TG), infrared spectroscopy (FTIR), and scanning electron microscopy (SEM) images. In addition, the EAE loading and kinetic release were determined. Finally, the effect of BC membrane containing *E. angustifolium* extract (BC-EAE) was evaluated against the selected HT-29 cancer cell line.

## 2 Materials and methods

### 2.1 Preparation and characterization of BC

#### 2.1.1 Plant material

The aerial part of *E. angustifolium* was collected during the massive blooming phase from the natural state in July in Poland (N 53°23′18″, E 14°28′56″). The plants were selected randomly from several different, closely located places. Six samples were harvested and combined into one collective sample. The plant material was dried at room temperature in a well-ventilated area to a constant weight (Vitalone and Allkanjari, 2018). Samples were stored in the plant material repository room (No. EAE-AM2021-05) at the Chair and Department of Cosmetic and Pharmaceutical Chemistry of the Pomeranian Medical University in Szczecin, Poland. The plant material was ground in the grinder and sieved using a circular-hole screen (8 mm mesh). Next, 5 g of dried raw material were extracted with 100 mL of 70% (v/v) ethanol (Chempur, Piekary Śląskie, Poland) for 60 min in an ultrasonic bath at a frequency of 40 kHz. Then, the obtained extracts were collected and filtered through Whatman filter paper No. 10, codified EEA03 (Merck, Darmstadt, Germany). After filtration, *E. angustifolium* extract was evaporated under reduced pressure at 40°C. A stock solution at 50 mg/mL concentration was prepared from the dried extracts in 70% (v/v) ethanol. The samples were stored in the dark at 4°C until placed in BC.

#### 2.1.2 BC production

*Komagataeibacter xylinus* bacteria strain from DSMZ—German Collection of Microorganisms and Cell Cultures (DSM 46604) was used for bacterial cellulose production. A buffered Hestrin-Schramm medium (bacto-peptone 5 g/L, yeast extract 5 g/L, di-sodium hydrogen phosphate 2.7 g/L, citric acid monohydrate 1.15 g/L) (Chempur, Piekary Śląskie, Poland) was used for the bacteria cultivation. First, all ingredients were dissolved in distilled water and then autoclaved at 121°C for 15 min. In the next step, a 50% (w/w) of the filter-sterilized aqueous glucose solution (Chempur, Piekary Śląskie, Poland) was added to the culture medium to reach a concentration of 20 g/L carbon source. Finally, the medium was buffered to reach a pH level of 5.0.

BC production was performed in plastic litter boxes (253 × 325 × 57 mm). First, a 1.2 L volume of the culture medium was placed in each litter box. Then, a 100 µL inoculum from the original stock sample was transferred to the medium. Afterwards, each litter box was secured with food foil and placed in an incubator (30°C) for 10 days. After incubation, the produced BC was harvested from the litter boxes. Each BC sheet was washed in distilled water to remove the remaining content of the medium and then immersed in a hot (80 °C) 0.1 M aqueous solution of sodium hydroxide for 30 min to remove any bacteria cells. Then, processed BC was rewashed in distilled water to remove NaOH remnants (pH = 7 of the solution reached).

#### 2.1.3 Preparation of BC containing *E. angustifolium* extracts (BC-EAE)

BC membranes were prepared by cutting off round pieces of 14.5 cm in a diameter, then weighed and manually pressed to remove 50%–60% of their water content. The dried BC membranes were soaked in ethanol solutions containing different concentrations of *E. angustifolium* extract*:* 0.25%, 0.5%, 1%, and 2.5% w/v for 24 h. The samples were incubated until the extract was entirely absorbed. The cellulose films were then dried at 40°C in a ventilated oven for 8 h. All membranes were subsequently weighed to determine the plant extract content. BC membranes for the biocompatibility study were prepared according to the same method without adding EAE. The membranes were stored in a desiccator until use.

#### 2.1.4 Fourier transform infrared-attenuated total reflectance (ATR-FTIR)

FTIR spectra were obtained in a Thermo Scientific Nicolet 380 spectrometer (Waltham, MA, United States of America) equipped with an ATR diamond plate. Thirty-two scans were acquired in the 4,000–400 cm^−1^ range with a resolution of 4 cm^−1^.

#### 2.1.5 Thermogravimetric analysis

Thermogravimetric analysis (TG) and its derivative (DTG) were carried out with TG 209 F1 Libra (Netzsch, Germany). All analyses were performed with a 5 mg sample in 6.8 mm (85 µL) crucibles under an air atmosphere between 25°C and 1,000°C, at a rate of 10 °C min^−1^. The analysis was conducted under oxidative conditions, with air and nitrogen flows of 25 cm^3^ min^−1^ and 10 cm^3^ min^−1^, respectively.

#### 2.1.6 Scanning electron microscopy (SEM)

The examination of the surface morphology was performed by Tescan Vega 3 scanning electron microscopy (SEM; Tescan, Brno, Czech Republik). SEM micrographs of all BC membrane surfaces were obtained operating at 30 kV. Samples were placed in steel support and coated with evaporated carbon. 2 samples of each membrane were analyzed.

#### 2.1.7 Accumulation of bioactive compounds in BC

The amount of total polyphenol content in the membranes was determined according to the modified method by Taokaew et al. (Taokaew et al., 2014), i.e., by immersing each sample (of 1 cm in diameter) in absolute ethanol and continuous stirring at 300 rpm at room temperature for 48 h. Afterward, the solutions were collected, and the amounts of the bioactive compounds in the films were determined. All samples were submitted for spectrophotometry analysis for total polyphenol content.

#### 2.1.8 Total polyphenol content determination

Total polyphenol content was determined with the Folin–Ciocalteu method as described previously (Nowak et al., 2019). Shortly, to 0.15 mL of the studied sample, 0.15 mL of tenfold diluted Folin–Ciocalteu reagent (Merck, Darmstadt, Germany), 1.35 mL of 0.01 M sodium carbonate (Chembur, Piekary Śląskie, Poland) solution and 1.35 mL of water were added and mixed. The cuvette was sealed with a stopper and then incubated for 15 min at room temperature. After this time, the spectrophotometric measurement was carried out at 765 nm. Three samples were prepared for each extract. Gallic acid (GA; Merck, Darmstadt (Germany) was applied as a standard, and results were expressed as gallic acid equivalents (GAEs) in mmol GA/dm^3^.

### 2.2 *In vitro* release studies

The following release tests were performed:

1) According to the modified Taokaew penetration method (Taokaew et al., 2014). In short, the release was evaluated using the Franz diffusion cells system (SES GmbH Analyse Systeme, Germany). Each BCs membranes: BC-2.5%EAE, BC-1%EAE, BC-0.5%EAE, and BC-0.25%EAE (a circular shape with a diameter of 1 cm) were placed on a donor compartment of the diffusion cells, covered with a dialysis tubing cellulose membrane (D 9777-100FT, Sigma Aldrich). Then 100 µL of PBS was added to each BCs and covered with microscope coverslips (Nowak et al., 2021c) (Silva et al., 2020). The acceptor chamber (of 8 mL volume) content was stirred with a magnetic stirring bar at the same speed for all the cells, and a constant temperature of 37.0°C ± 0.5°C *via* a thermostat (VEB MLW Prüfgeräte-Werk type of 3,280) was maintained. The experiment was carried out for 96 h. The samples were collected after 0.5; 1; 3; 5; 8; 24; 28; 48; 72, and 96 h of stirring. After indicated time, the acceptor fluid (0.5 mL) aliquots were withdrawn and refilled with fresh PBS. Then all samples were submitted for spectrophotometry analysis for total polyphenol content determination.
2) Directly from tubes according to the previously described method (Cacicedo et al., 2016). Shortly, BC membranes (circularly shaped of 1 cm in diameter) were placed in 1.0 mL of PBS or cell culture medium and thermostated at 37°C. The samples were collected after 0.5; 1; 3; 5; 8; 24; 28; 48; 72 and 96 h. Afterwards, the acceptor fluid (0.5 mL) aliquots were withdrawn and refilled with fresh buffer (PBS/medium). The release readings were acquired from three independent experiments. Then, all the samples were submitted for spectrophotometry analysis for total polyphenol content.

The spectrophotometric results (OD) from both methods were then normalized to the polyphenol concentration in BC-EAE determined in the eluate after continuous stirring of BC (Ø = 1 cm) in absolute ethanol for 48 h (assumed as total concentration, maximal possible release).

$$Polyphenol\;released=\left[OD\left(sample\right)/OD\left(total\;concentration\right)\right]*100\%$$ (1)

### 2.3 Cell culture study

#### 2.3.1 Anticancer activity of *E. angustifolium* (screening study)

The cytotoxicity of *E. angustifolium* against selected (based on literature reports) cancer cell lines and normal human fibroblasts was evaluated. Malignant melanoma cells (A375, ECACC 88113005), human breast adenocarcinoma cells (MCF7, ECACC 86012803), colon adenocarcinoma (HT-29, ECACC 91072201), human Caucasian lung carcinoma (A549, ECACC 86012804) and human Caucasian hepatocyte carcinoma (HepG2, ECACC 85011430) were purchased from European Collection of Authenticated Cell Cultures (ECACC). The human dermal fibroblast (HDF) cells derived from the skin were isolated according to the previously described protocol (Nowak et al., 2022), approved by the Ethical Committee of Pomeranian Medical University in Szczecin (KB-0012/02/18-A). All cells were cultured in standard conditions (5% CO_2_, 37°C) in recommended culture medium supplemented with heat-inactivated fetal bovine serum (FBS, EURx, Gdansk, Poland), L-glutamine (2 mM, Sigma-Aldrich Merck Group, St. Louis, MO, United States of America) and penicillin-streptomycin (Sigma-Aldrich Merck Group, St. Louis, MO, United States of America). Cell lines were routinely tested for the presence of mycoplasma.

The cell viability was evaluated using a PrestoBlue™ HS Cell Viability Reagent (Thermo Fisher Scientific, United States of America) based on the reduction of resazurin to highly fluorescent resorufin, which amount is directly correlated with the number of viable proliferating cells. Tested cells were seeded in 96-well black microplates (Greiner, Austria) at a varied density, adjusted to the doubling time of individual cell lines, and left to adhere for 24 h. Afterwards, the cell culture medium was removed and replaced with 90 µL of the fresh medium containing 500, 250, 125, 62.5, and 31.25 μg/mL of EAE (previously sterilized using membrane filters 0.22 µm). The final ethanol concentrations did not exceed 1%, and its effect on cell viability was also evaluated. The cells without tested compounds were used as controls, and the tested extracts in a medium without cells as a blank. After 48 h of treatment, PrestoBlue reagent (10 μL) was added to each well and incubated for 30 min. The fluorescence was measured using a spectrophotometric microplate reader (Infinite 200 Pro, Tecan, Switzerland) at ex/em: 560/594. The results were normalized to the control (100% viability) according to the following formula:

$$The\;cell\;viability=\frac{\left({\mathrm{RFU}}_{test}-{\mathrm{RFU}}_{blank}\right)}{\left({\mathrm{RFU}}_{control}-{\mathrm{RFU}}_{blank}\right)}100\%$$ (2)

where: ${\mathrm{RFU}}_{test}$ - relative fluorescence units of cells with a medium containing the extracts ${\mathrm{RFU}}_{blank}$ - relative fluorescence units of cell-free medium with the respective extract ${\mathrm{RFU}}_{control}$ - relative fluorescence units of cells with free medium

The readings were obtained from at least three independent experiments (each conducted in triplicate). In addition, the IC_50_ values (the inhibitory concentration causing 50% growth inhibition) were evaluated using an online calculator (AAT Bioquest, Inc., Quest Graph™ IC50 Calculator (v.1), retrieved from: https://www.aatbio.com/tools/ic50-calculator-v1 (accessed on: 10 June 2022).

#### 2.3.2 Direct cytotoxicity assay of BC/BC-EAE

Cytotoxicity assay was performed according to the previously described protocol by Orlando et al. (Orlando et al., 2020), and it complied with the ISO 10993-5 standard for the test by direct contact. The BC-EAE, as well as empty BC in duplicates, were punched into circular sheets of 5.4 mm in diameter, sterilized by autoclaving at 126 °C for 11 min, and aseptically transferred to HT-29 cells, which had been seeded in a 24-well plate (2 × 10^4^ cells/well and 3 × 10^4^ cells/well), the day before. The cell culture medium (0.5 mL) had been replaced directly before the membrane placement. The cells in a free medium (without BC) were used as the control. After 48 and 72 h incubation in cell culture conditions, the BC-EAE, blank BC, and the medium were removed, and 0.4 mL of fresh medium containing 10% v/v of PrestoBlue™ HS Cell Viability Reagent (Thermo Fisher Scientific, United States of America) was added to each well and then incubated in the same conditions for 30 min. Afterwards, 0.3 mL (3 × 0.1 mL) of the solution from each well was transferred into 96-well black microplates (Greiner, Austria), and fluorescence was measured as described above. The readings were acquired at five independent experiments.

#### 2.3.3 Apoptosis assay

HT-29 cells were seeded in 24-well plates (2 × 10^4^ cells/well and 3 × 10^4^ cells/well). After 24 h, the cell culture medium was removed, and then the fresh medium with BC-EAE/empty BC was added and incubated for another 48/72 h. Untreated cells were used as the control. After incubation, culture medium and PBS (used to wash the cells) were colleceted, and cells were harvested by trypsinization. After centrifugation, the supernatant was discarded, and the cells pellet was resuspended in equal amount of medium and the Muse Annexin V & Dead Cell Reagent (Luminex Corporation, Austin, TX, United States of America), mixed thoroughly, and stained for 20 min (at room temperature in the dark). Samples were analyzed by Muse Cell Analyzer (Luminex Corporation, Austin, TX, United States of America). The assay relies on the detection of phosphatidylserine (PS) on the surface of apoptotic cells by binding with Annexin V-PE. A dead cell marker (7-AAD) is also used as an indicator of cell membrane structural integrity, because it is excluded from live and healthy cells, permeates the late-stage apoptotic and dead cells. Simultaneous staining with these two dyes allows to distinguish of four populations of cells: viable (Annexin V-PE− and 7-AAD−), early apoptotic (Annexin V-PE+ and 7-AAD−), late apoptotic/already dead cells (Annexin V-PE+ and 7-AAD+), dead cells (Annexin V-PE− and 7-AAD+). According to the manufacturer’s protocol (based on the cell size index), debris was excluded from further analysis. The readings were acquired from three independent experiments.

#### 2.3.4 Microscopy imaging

Additionally, optical microscopy imaging of the tested cells was performed just prior to PrestoBlue/apoptosis analysis of harvested cells using Smart Fluorescent Cell Analyzer Microscope JuLi (Seoul, Korea).

### 2.4 Statistical analysis

The results are presented as mean ± standard deviation (SD). Statistical analysis was carried out using Statistica 13.3 (StatSoft Inc., Tulsa, OK, United States of America) and Student’s t-test or ANOVA test followed by a *post hoc* Tukey’s multiple comparisons test. A *p*-value level of <0.05 was considered statistically significant.

## 3 Results

### 3.1 Characterization of BC-EAE

#### 3.1.1 ATR-FTIR spectra

**FIGURE 1:**
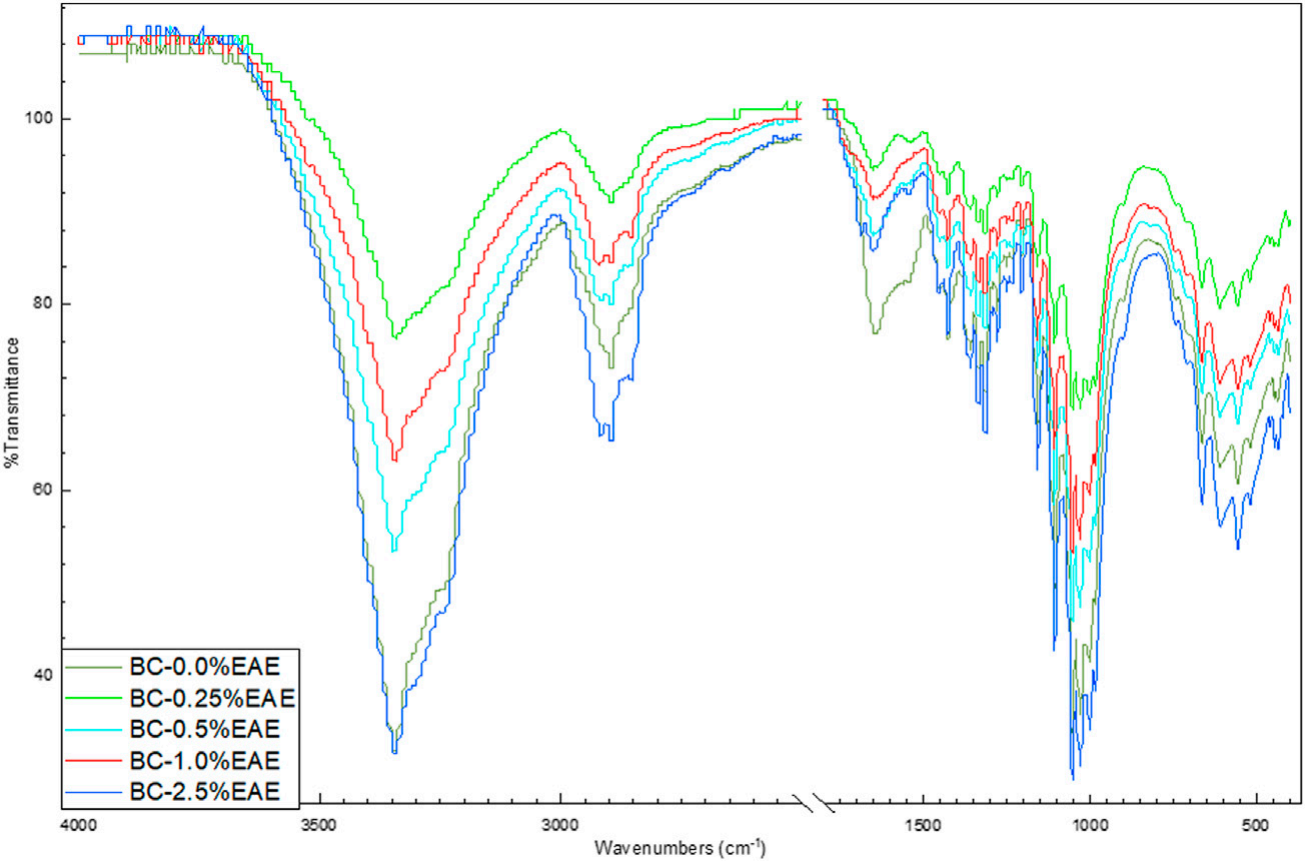
ATR-FTIR spectra of bacterial cellulose—BC (violet), BC-0.25%EAE (light blue), BC-0.5%EAE (green), BC-1%EAE (red), and BC-2.5%EAE (dark blue).

ATR-FTIR spectra were carried out to confirm that the materials are cellulose-based. The FTIR spectra of BC, BC-EtOH, BC-0.25%EAE, BC-0.5%EAE, BC-1%EAE, and BC-2.5%EAE are shown in Figure 1. Single FTIR-ATR spectra are presented in the Supplementary material (Supplementary Figures S1–S5). All obtained FTIR spectra presented typical absorption bands specific for cellulosic materials. The groups O-H, C-H, and C-O-C are visible at 3,345, 2,896, and 1,158 cm^−1^, respectively. Furthermore, the weak and broadband centered at approximately 900 cm^−1^ and strong band centered at approximately 1,425 cm^−1^, which can be assigned to CH_2_ bending vibration, defined the cellulose as cellulose I, which suggested that BC produced in this study could be pure cellulose. The spectra of BC-EAE are very similar to the pure BC spectrum. The FTIR spectra collected at different points of the surface and inner layers of EAE-loaded membranes showed a similar profile.

#### 3.1.2 TG analysis

**FIGURE 2:**
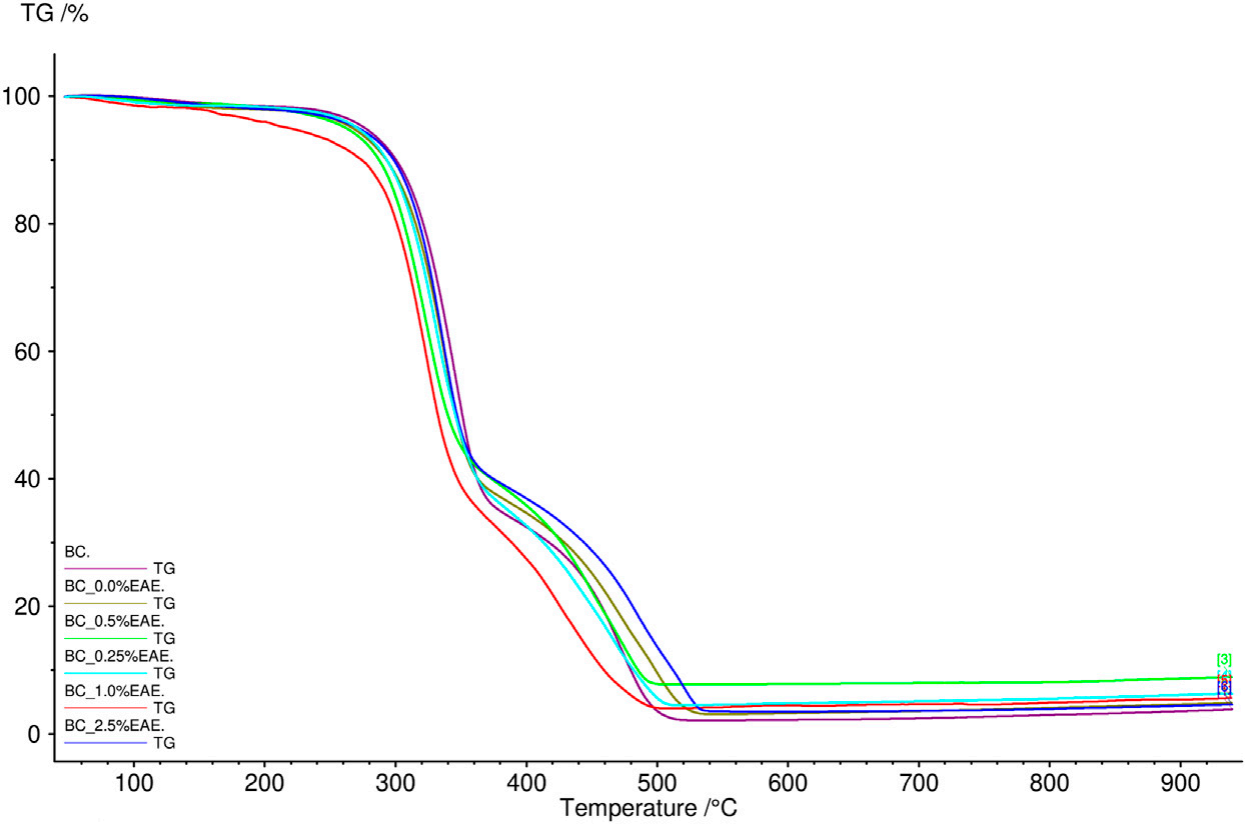
TG curves of bacterial cellulose—BC (violet), BC-0.25%EAE (light blue), BC-0.5%EAE (green), BC-1%EAE (red), and BC-2.5%EAE (dark blue).

**FIGURE 3:**
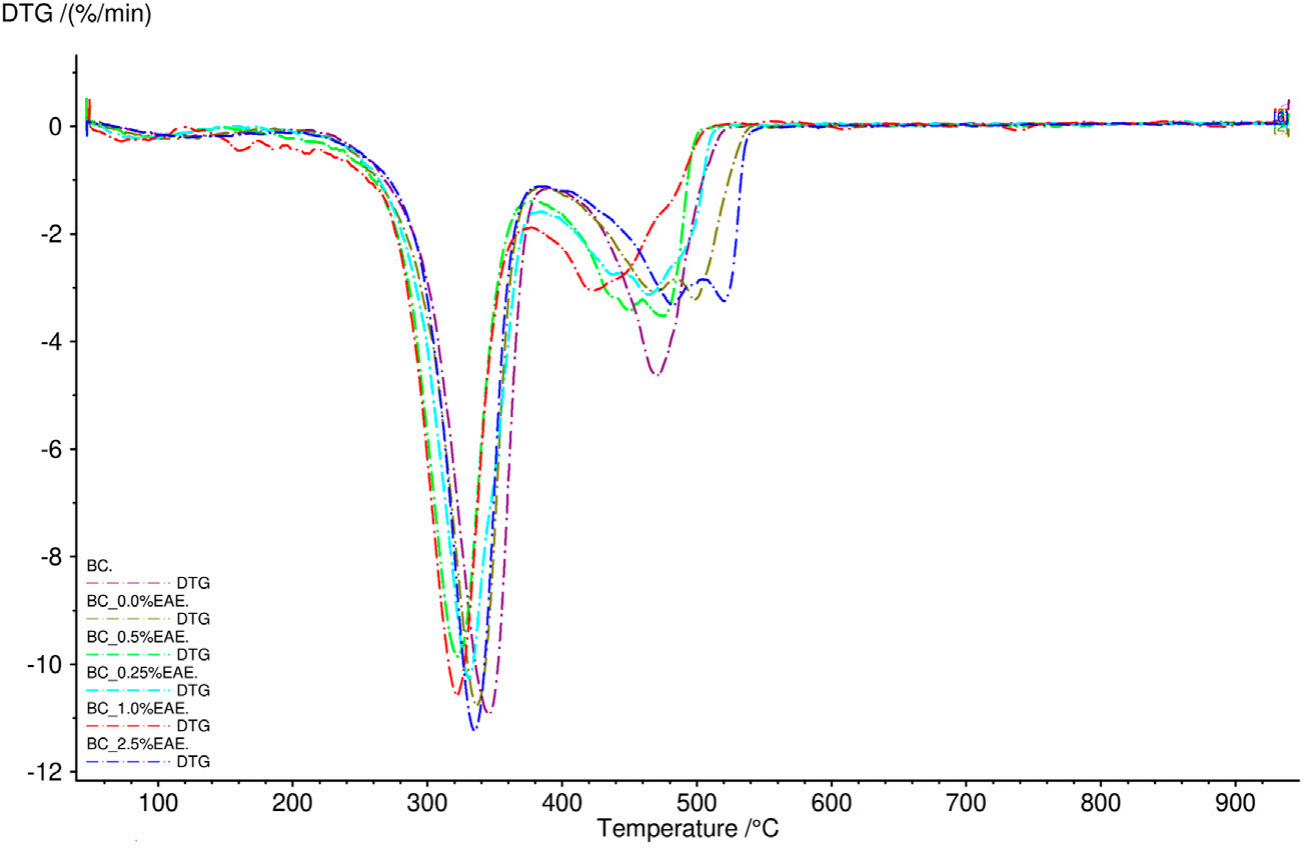
DTG curves of bacterial cellulose—BC (violet), BC-0.25%EAE (light blue), BC-0.5%EAE (green), BC-1%EAE (red), and BC-2.5%EAE (dark blue).

The thermal stability of the obtained cellulose membranes was also determined. For this purpose, TG analysis was used. The obtained results are presented in Figures 2, 3 and Supplementary Figures S6–S11 (TG and DTG curves). As can be seen, the differences between the individual cellulose membranes are small. Two clearly visible distinct steps were recorded for the weight loss of the BC membranes, namely, the weight loss of around 280°C and 320°C. All obtained membranes were very stable, showed no degradation up to 250°C, and had a comparable residual mass.

#### 3.1.3 SEM

**FIGURE 4:**
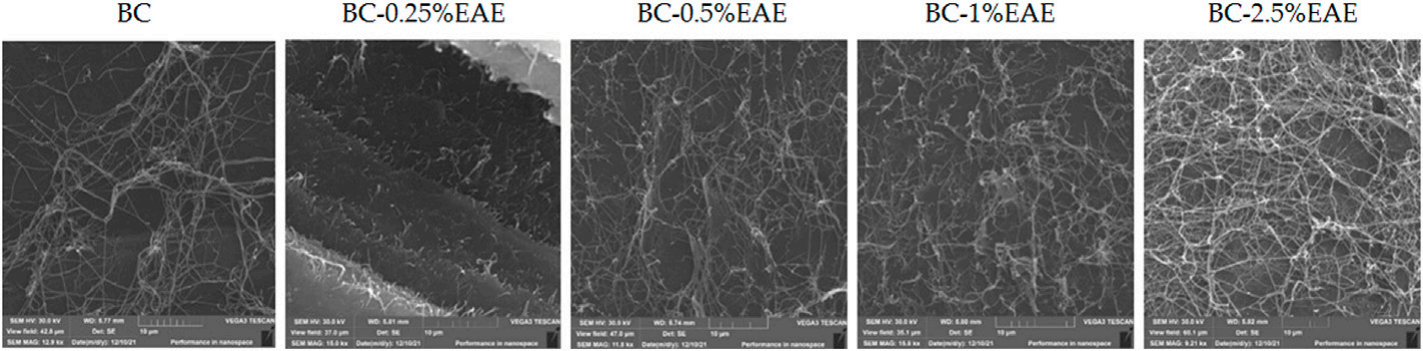
The SEM micrographs of BC, and BC containing EAE at different concentrations on a scale of 10 µm.

The SEM images of the dried BC and BC-EAE are presented in Figure 4. The SEM image shows that the BC surface consists of many pure nanofibers that form an aggregated structure. A characteristic three-dimensional network was observed in all membranes. However, in the case of BC containing EAE, denser and longer fibers were visible. This is probably due to the high concentration of plant compounds in the extract and the deposition of active substances inside the membrane.

#### 3.1.4 Accumulation of bioactive compounds in BC

**FIGURE 5:**
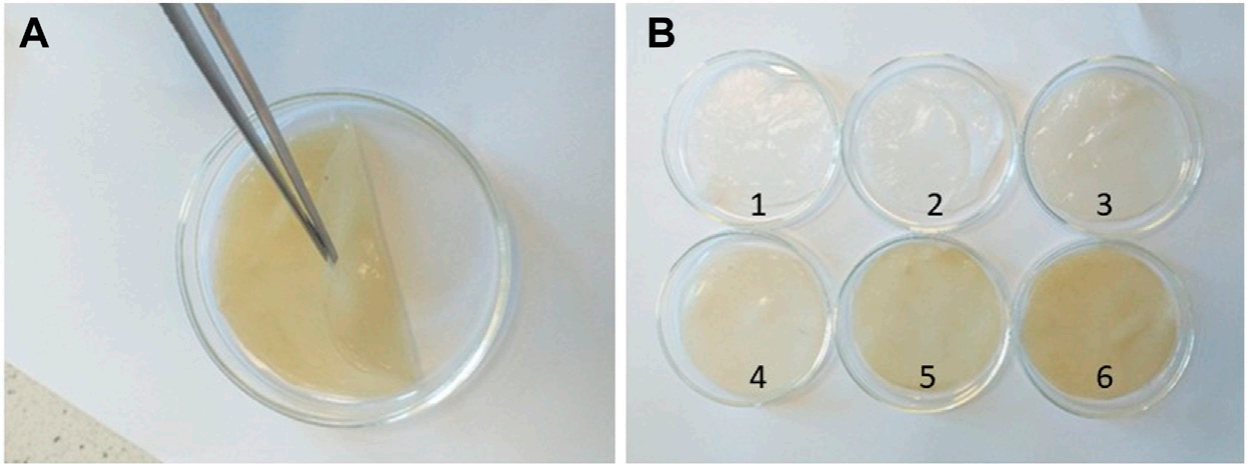
Image of a single BC before drying **(A)** and visual difference between the tested BC **(B)**: BC (1), BC-EtOH (2), BC-0.25%EAE (3), BC-0.5%EAE (4), BC-1%EAE (5) and BC-2.5%EAE (6).

In order to produce BC-EAE, empty membranes were incubated in ethanol solutions containing different concentrations of *E. angustifolium* (0.25%, 0.5%, 1%, 2.5% w/v). The obtained membranes were homogeneous, as shown in Figure 5. The crude EAE content and total polyphenol accumulation (as the main bioactive compounds) in each BC-membranes were evaluated and shown in Table 1. The results confirmed the efficient absorption of plant extracts into the BC, and the content of bioactive compounds increased proportionally to the concentration of the extract.

**TABLE 1:** The plant extract content and total polyphenol accumulation in BC-EAE.

Sample	mg EAE extract/g membrane*	Total polyphenol concentration** (mmol/dm^3^)
BC	-	nd
BC-EtOH	-	nd
BC-0.25%EAE	54.8	0.32 ± 0.02
BC-0.5%EAE	129.5	0.61 ± 0.03
BC-1%EAE	219.1	1.23 ± 0.08
BC-2.5%EAE	434.1	2.26 ± 0.16

nd-not detected.

* Calculated after membrane drying at 40 °C in a ventilated oven for 24 h.

**Determined in the eluate after continuous stirring of BC (Ø = 1 cm) for 48 h according to the modified Taokaew et al. method (Taokaew et al., 2014).

### 3.2 Release study

**FIGURE 6:**
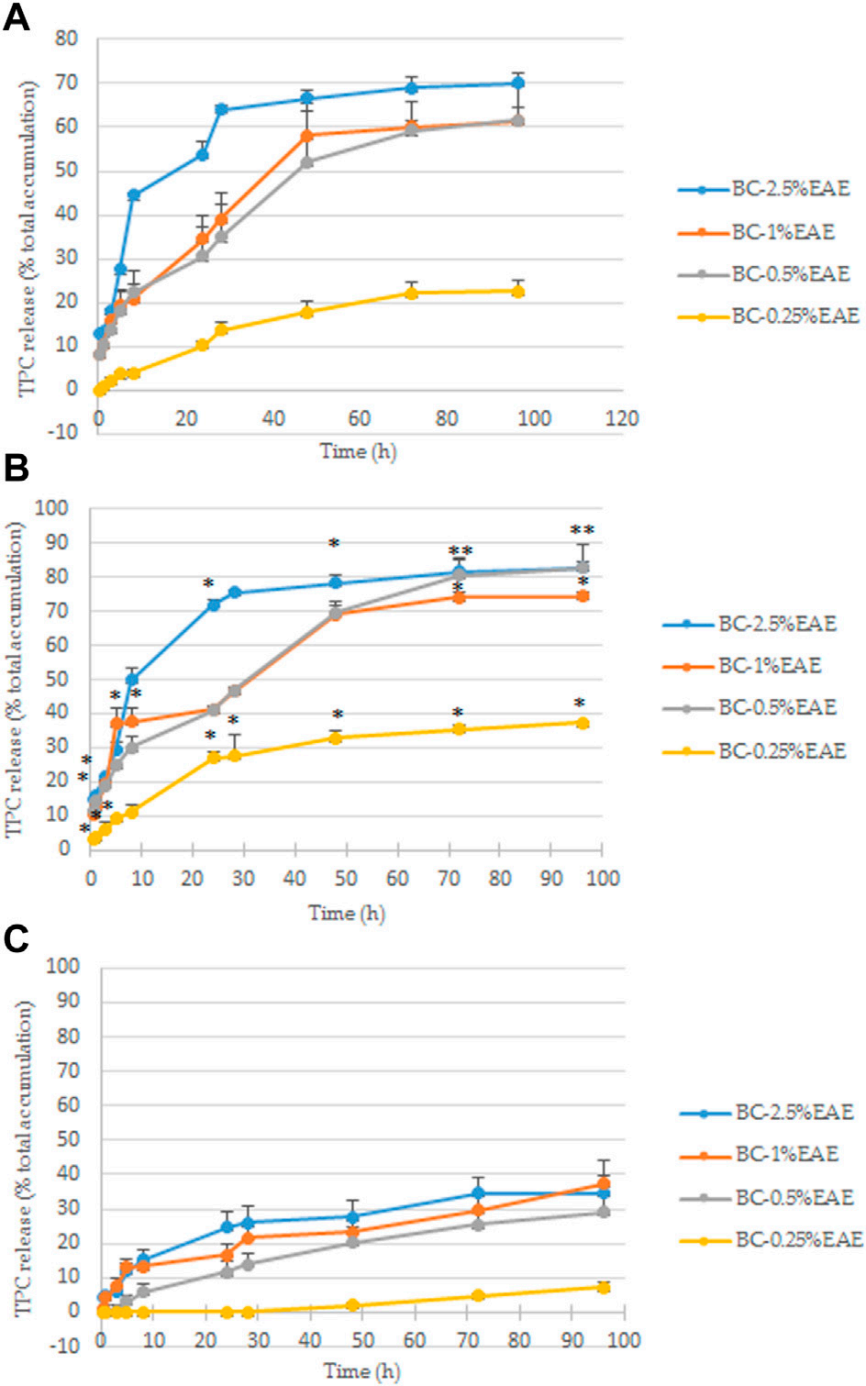
The total polyphenol content released from BC at 37°C in PBS, determined according to the modified Taokaew method (Taokaew et al., 2014) **(A)** and the Cacicedo method (Cacicedo et al., 2016) **(B)**; statistical differences between these two method were determined by Student’s t-test and presented as *p < 0.05. The total polyphenol content released from BC at 37 °C in the cell culture medium was determined according to the modified modified Cacicedo et al., 2016 **(C)**. The differences between these two fluids (PBS and medium, Fig. B and C) determined by Student’s t-test, were significant at each point but for the sake of clarity are not shown on the chart. All findings are presented as a percentage of total polyphenol content accumulated in BCs (Table 1).

The release of the plant extract from the BC-EAEs in PBS was evaluated by two different methods, where the total polyphenol content was measured. Both methods are often used during studies on the release of active substances from drug carriers. However, the first one (based on Franz diffusion cells system) is most often preferred in research on transdermal drug release systems, the second for locally or systemically administered drug carriers. Due to the possibility of using membranes as carriers for transdermal and local delivery system, both methods were used. As shown in Figures 6A,6B, the most significant differences between those two methods were shown at the extreme concentrations (BC-2.5%EAE and BC-0.25%EAE). However, regardless of the method used, the release rates of the polyphenols apparently depended on the initial extract amount in the membrane. Higher initial concentrations of the extract in BC led to a higher release rate. For instance, according to Franz diffusion studies, after 96 h, total polyphenol content released from BC-2.5%EAE, BC-1%EAE, BC-0.5%EAE, and BC-0.25%EAE were equal to 70.17% ± 2.06%, 61.44% ± 2.95%, 61.54% ± 8.15% and 22.46% ± 2.65%, respectively (Figure 6A). The overlapping curves of BC-1%EAE and BC-0.5%EAE indicate a similar release profile. The initial release of the bioactive compounds from BC containing higher extract content was very rapid during the first 48 h. In the case of BC-2.5%EAE, the release burst was the most striking and reached 66.51% ± 1.86% and 78.33% ± 2.19% after 48 h, according to Taokaew (Taokaew et al., 2014) and Cacicedo (Cacicedo et al., 2016) release determination method, respectively. After this time, the release decline became slow and linear in all kinetics.

Contrarily, the release rates of bioactive compounds to the cell culture medium were relatively low for the entire time of the experiment and devoid of the characteristic burst (Figure 6C). The maximum release of polyphenol from BC-2.5%EAE, BC-1%EAE, BC-0.5%EAE, BC-0.25%EAE yielded 34.59% ± 4.86%, 37.30% ± 6.65%, 28.99% ± 5.40% and 7.21% ± 1.30%, respectively at 96 h. Similarly to the PBS analysis, the release of polyphenols from BC with the lowest plant extract content (BC-0.25%EAE) was much slower.

### 3.3 Anticancer activity of *E. angustifolium*

**FIGURE 7:**
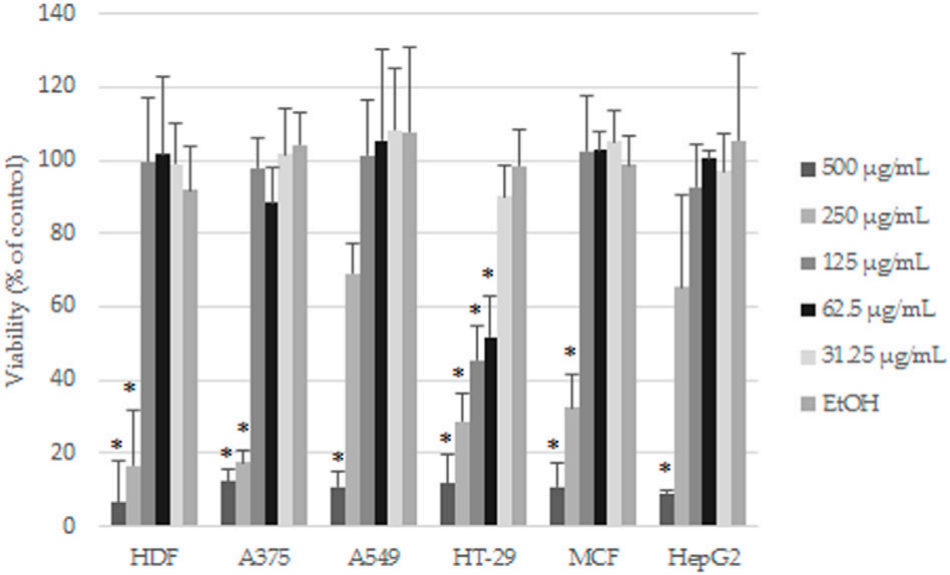
Effects of EAE on the cells viability after 48 h of treatment. The results are expressed as mean and SD from three independent experiments; *p < 0.05 vs. control (untreated cells) (Student’s t-test).

Cytotoxicity of the EAE was evaluated using a resazurin-based cell viability assay. Five increasing concentrations of the extract (31.25–500 μg/mL) were tested in normal human fibroblasts (HDF) and five cancer cell lines selected from literature reports. After 48 h of incubation, the reduction of cell viability in a concentration-dependent manner was noticed (Figure 7). The highest tested concentrations (250 and 500 μg/mL) markedly decreased the cell viability of all tested cells (including HDF), whereas, at lower concentrations (between 31.25 and 125 μg/mL), except in the case of HT-29, cell viability was comparable to controls. The obtained results were consistent with the microscopy imaging. Supplementary Figure S12 show images of the control and cancer cells treated with the highest concentrations of EAE, where the differences in viability were the most striking. At EAE concentrations of 250 and 500 μg/mL, only dead or morphologically changed (in the case of HDF) cells were observed. The HT-29 cell line was unique, where the differences in the cell morphology between EAE (at concentration 125 μg/mL) and controls were still noticeable. As shown in Table 2, according to IC_50_ values, HT-29 cells were the most sensitive to EAE (61.73 ± 6.42 µM), whereas A549 and HepG2 showed the lowest sensitivity, with nearly five times higher IC_50_ values.

**TABLE 2:** The IC_50_ values (μg/mL) were determined using PrestoBlue assay after 48 h of treatment. Data are expressed as mean ± SD from at least three independent experiments.

Cell line	HDF	A375	A549	HT-29	MCF7	HeG2
IC_50_	191.81 ± 44.31	204.78 ± 26.81	295.42 ± 55.09	61.73 ± 6.42	214.56 ± 27.96	301.86 ± 77.33

### 3.4 Direct cytotoxicity assay of BC-EAE

**FIGURE 8:**
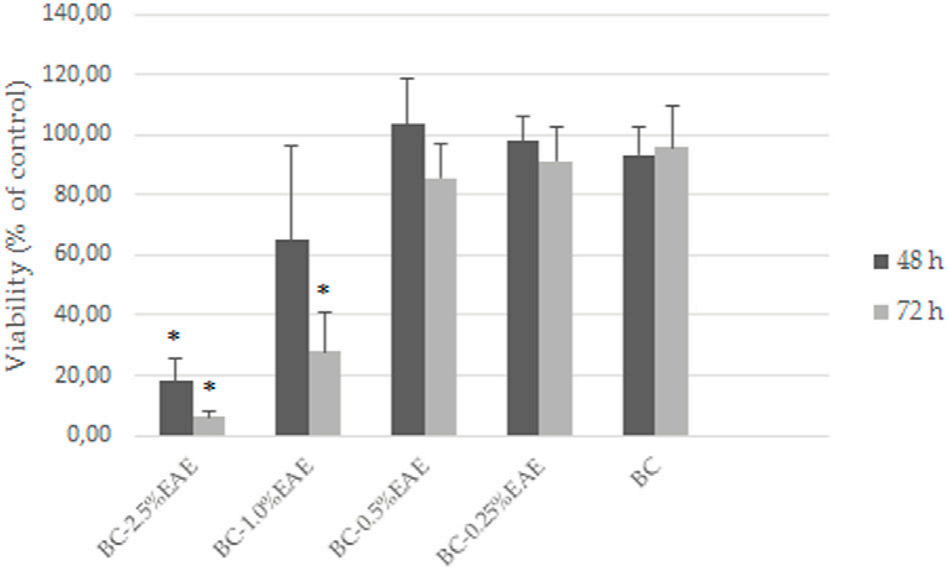
The viability of HT-29 cells after exposure to BC-EAE for 48 h and 72 h; *p < 0.05, statistical significance compared with BC (Student’s t-test).

**FIGURE 9:**
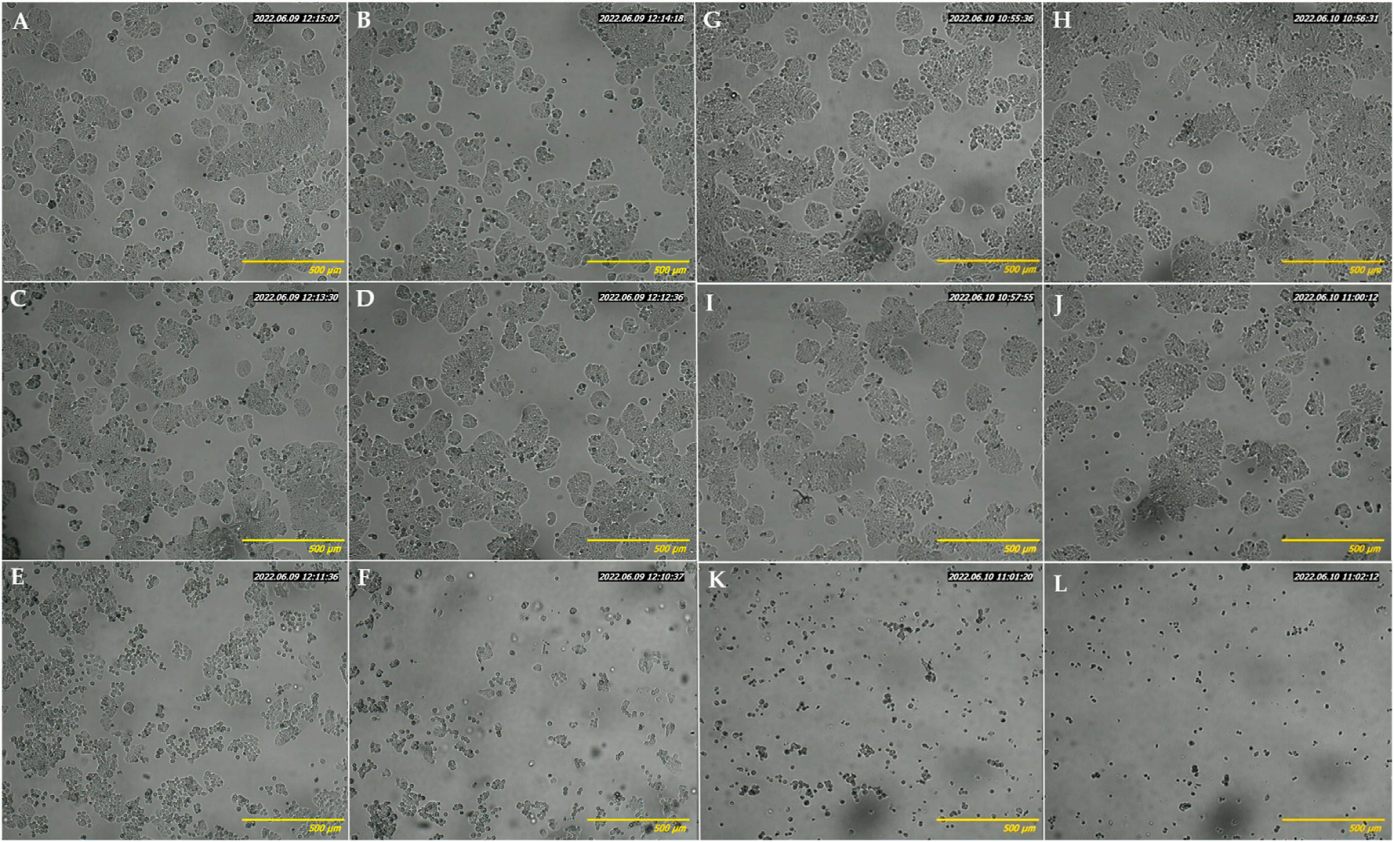
Optical microscopy images of HT-29 cells after 48 h **(A–F)** and 72 h **(G–L)** incubation with medium **(A, G)**, empty BC **(B, H)**, BC-0.25%EAE **(C, I)**, BC-0.5%EAE **(D, J)**, BC-1%EAE **(E, K)**, BC-2.5%EAE **(F, L)**.

HT-29 cells, the most sensitive to the above-tested crude extract, were selected to evaluate the effect of increasing concentrations of EAE absorbed into the BC. Firstly, the PrestoBlue assay results demonstrated the biocompatibility of empty BC (with cell viability of nearly 100%) after 48 and 72 h of treatment (Figure 8). Contrary to the unmodified BC, BC-EAE revealed time and dose-dependent cytotoxicity against cancer cells. The maximum and statistically significant cell inhibitory effect was reached after exposure to BC-2.5%EAE. The EAE release was analyzed in parallel with the cytotoxicity study regarding the total polyphenol content as the main bioactive compound (Table 3). Namely, after 48 and 72 h of treatment, the plant extract released from BC-2.5%EAE (corresponding to 0.27 ± 0.08 and 0.40 ± 0.05 mmol/dm^3^) reduced cell viability to 18.16% ± 7.80% and 6.15% ± 2.11%, respectively. In comparison, BC-1.0%EAE decreased cell viability to 65.02% ± 31.27% and 27.92% ± 12.77% after 48 h and 72 h exposure, respectively. In the case of BC-1.0%EAE, the observed cytotoxicity was statistically significant only after 72 h of treatment, when the concentration of the released polyphenols in the medium reached 0.26 ± 0.02 mmol/dm^3^. No significant differences in cell viability between BC-0.25%EAE, BC-0.5%EAE, and empty BC were observed. The obtained results were consistent with microscopy imaging (Figure 9). The most pronounced differences between controls (Figures 9A,G) and treated cells were observed at BC-1.0%EAE and BC-2.5%EAE, especially after 72 h of the treatment (Figures 9K,L). The cancer cells cultured with BC-1.0%EAE and BC-2.5%EAE exhibited a disturbed rounded cell shape after 48 h (Figures 9E,F), and only dead cells were visible after prolonged incubation (72 h). After 72 h, single dead cells were also observed in samples incubated with BC-0.5%EAE (Figure 9J).

**TABLE 3:** The total polyphenol concentration (mmol/dm3) released from BC-EAE to the medium after 48 and 72 h.

	BC-0.25%EAE	BC-0.0.5%EAE	BC-1%EAE	BC-2.5%EAE
48 h	0.01 ± 0.03	0.05 ± 0.04	0.12 ± 0.05	0.27 ± 0.08
72 h	0.12 ± 0.04	0.16 ± 0.03	0.26 ± 0.02	0.40 ± 0.05

### 3.5 Apoptosis assay

**FIGURE 10:**
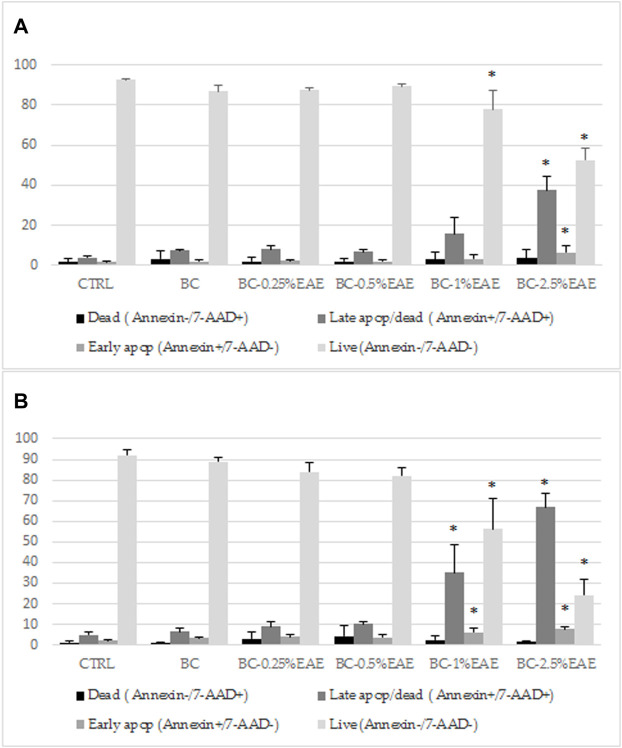
The effect of indicated compounds on the induction of apoptosis in HT-29 cells after 48 h **(A)** and 72 h **(B)** of treatment, subsequently stained with Annexin V-PE and 7-AAD and analyzed by flow cytometry. Data are expressed as mean and SD from three independent experiments; *p < 0.05 vs. ctrl (ANOVA test followed by a *post hoc* Tukey’s multiple comparisons test).

**FIGURE 11:**
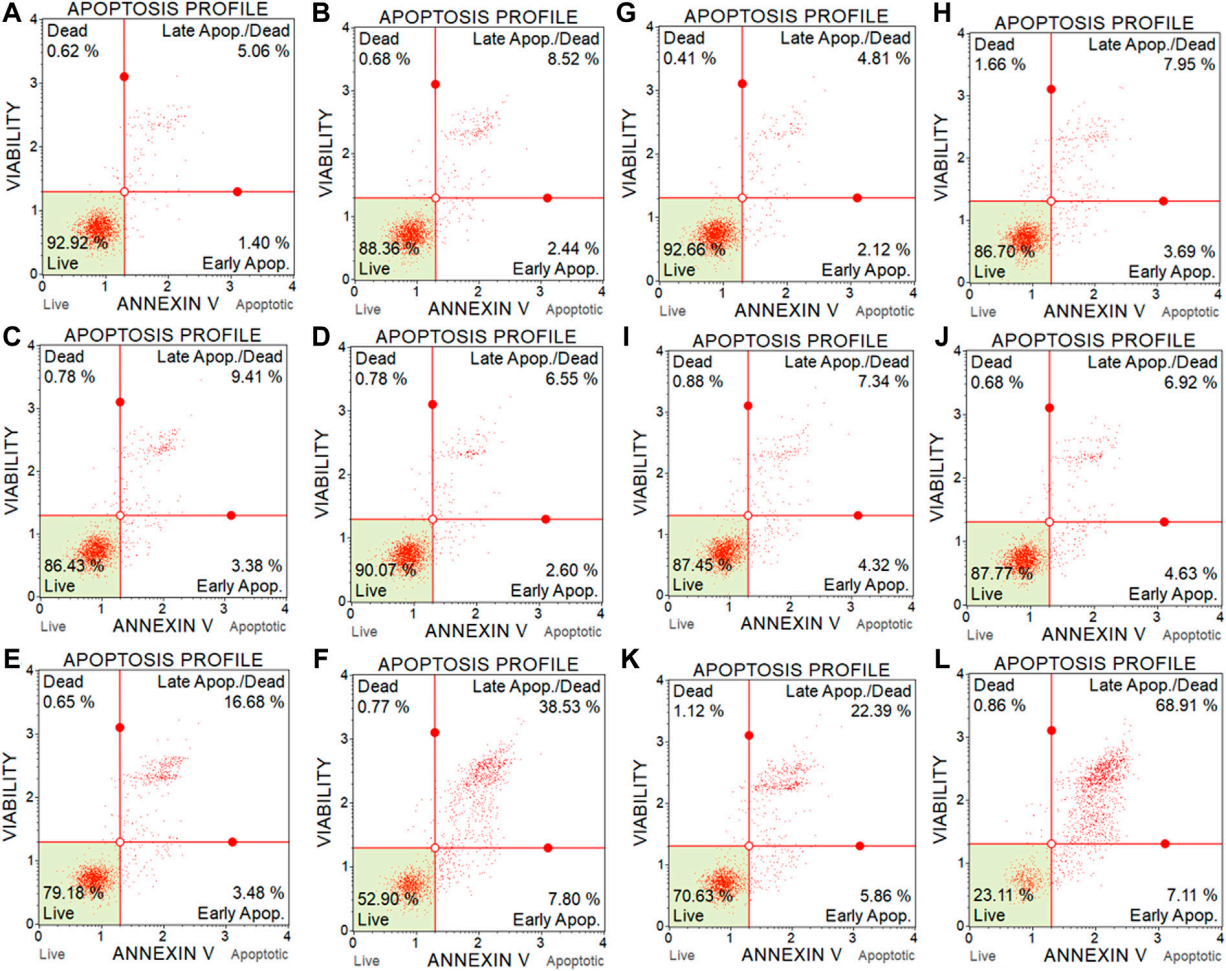
Representative flow cytometry scatter diagrams of HT-29 cell apoptosis after 48 h **(A–F)** and 72 h **(G–L)** incubation with medium **(A** and **G)**, with empty BC **(B** and **H)**, BC-0.25%EAE **(C** and **I)**, BC-0.5%EAE **(D** and **J)**, BC-1%EAE **(E** and **K)**, BC-2.5% EAE **(F** and **L)**.

The cytotoxicity results of BC-EAE were consistent with the apoptosis assay performed under the same conditions. As shown in Figure 10, the percentage of apoptotic cells was significantly increased in a dose and time-dependent manner. The population of apoptotic (both early and late) HT-29 cells significantly increased after 48 h (Figure 10A) of treatment with BC-2.5%EAE, whereas after 72 h (Figure 10B), also BC-1%EAE produced apoptosis of the neoplastic cells. After 48 h of treatment, the level of late apoptotic/dead cells (upper-right square in Figures 11A–F) increased from about 4.05% ± 0.85% (control cells) to 15.95% ± 7.85% and 37.53% ± 6.86% for BC-1%EAE and BC-2.5%EAE, respectively. After 72 h of incubation, the number of late apoptotic/dead cells increased from about 4.82% ± 1.59% (control cells) to 35.13% ± 13.51% and 66.90% ± 6.48% for BC-1%EAE and BC-2.5%EAE, respectively. No significant differences in cell viability between BC-0.25%EAE, BC-0.5%EAE, BC, and control were observed.

## 4 Discussion

Although intravenous injection is the most common route of anticancer drug administration, in some cases, direct delivery of chemotherapeutic agents to tumour tissues constitutes an alternative that may allow for the improvement of therapy outcomes. In addition, systemic application of anticancer agents produces risks of systemic side effects, which can be avoided by local drug delivery systems. In the current study, BC membranes were applied as a matrix for local delivery of the plant extract with potential anticancer activity. A simple immersion of BC in an ethanol solution of *E. angustifolium* (at concentrations of 0.25%, 0.5%, 1%, and 2.5%) has been applied as the most common and cost-effective loading method of active compounds in BC (Swingler et al., 2021). It has been proved that the plant extracts were effectively absorbed into the BC, proportionally to the increasing extract concentration. Namely, the cumulative amount of crude EAE absorbed into BC-0.25%EAE, BC-0.5%EAE, BC-1%EAE, and BC-2.5%EAE were found to be 54.8, 129.5, 219.1, 434.1 mg/g of the membrane, respectively. It should be pointed out that the total polyphenol contents, as the main bioactive compounds, increased parallelly to the plant extract concentrations. This data confirmed that the use of membranes allowed local delivery of high amounts of extracts, which could not be produced by systemic administration due to potential side effects.

The FTIR spectrum confirmed the BC purity and the presence of plant extracts. In addition, the FTIR analysis of bacterial cellulose shows typical absorption bands characteristic of cellulosic materials. As in our previous research, the bands in the range of 1,500–1,750 cm^−1^ were observed, which can be attributed to the C=O stretching vibration or the C=C stretching vibration, most probably from the compounds presented in EAE, such as tetrahydrogeranyl acetone, methyl palmitate, methyl oleate, glyoxylic acid, and 8-octadecenal (Nowak et al., 2021c). Furthermore, the FTIR spectra collected at different points of the surface and inner layers of the EAEs-loaded membranes showed a similar profile, confirming good dispersion of the extract ingredients inside the membranes.

In TG analysis, two distinct steps were recorded for the weight loss of the BC membranes (around 280°C and 320°C). The weight loss at 320°C was probably caused by cellulose degradation processes, such as depolymerization, dehydration, and decomposition of glucosyl units, followed by the formation of charred residues. Other authors observed maximum rates of weight loss for bacterial cellulose to occur at 360–390°C (Surma-Ślusarska et al., 2008) (Mohammadkazemi et al., 2015). The differences in thermogravimetric results may depend on several factors, such as sample preparation, sample size, morphology, and homogeneity (Kumbhar et al., 2015). Furthermore, all studied membranes were stable and resistant to degradation in temperatures up to 250°C.

The obtained membranes were homogeneous, indicating a good dispersion of EAE inside the tridimensional nanofibrillar network of BC. The uniformity of BCs was further confirmed by the surface SEM analysis. The surface of BC was composed of many fibrils and formed an aggregated structure, which could be observed in the SEM image. The high compactness of the BC fibrils is associated with the nature and amount of available carbon source, amount of inoculum, type of strain, and culture conditions, as well as the post-synthesis processing and drying method. The structure as well as the presence of pores of different sizes, provide an ideal environment for penetration of different materials, including plant extracts (Fatima et al., 2021). The nanofibrillars of BC containing plant extracts became longer and denser, especially after incubation with BC-2.5%EAE. This is probably due to the higher concentration of *E. angustifolium* in the extract and more intense deposition of the active substances inside the membrane. When plant extracts are incorporated into cellulose membranes of bacteria, the plant molecules penetrate deeply and fill BC pores. In the studies conducted by other authors, an increase in BC thickness was observed when mango extract (Taokaew et al., 2014) or papain (Asanarong et al., 2021) were incorporated. It should be also pointed that our previous study confirmed that the mechanical properties (measured by Young’s modulus, tensile strength, and elongation at break) of BC with and without EAE are comparable (Nowak et al., 2021b).

Our study confirmed that a higher initial amount of plant extract in BC correlated with a faster and more intensive release of polyphenols, the main bioactive compounds of the extract. Moreover, the release kinetics profile differed depending on the acceptor fluid. First of all, BC-2.5%EAE, BC-1%EAE, and BC-0.5%EAE incubated in PBS showed the highest release rate within the first 48 h, followed by a linear phase. Otherwise, the release rates of polyphenols to the culture medium were relatively low for the entire length of the experiment, providing the sustained release of active compounds in cell culture conditions. For instance, the maximum release of polyphenols from BC-2.5%EAE yielded about 70.2%–82.9% (depending on the method used) and 34.6% in PBS and medium, respectively. Contrary to our study, Taokaew et al. (Taokaew et al., 2021), who tested BC films containing mangosteen peel extract, showed that different media (water, DMEM, MEM) did not affect release rates of polyphenols, whereas decreased the release rate of α-mangostin. The authors supposed that the observed differences might be related to chemical properties of released compounds (i.e., hydrophilicity and molecular mass). Membrane nanostructure can also determine the release behavior of active substances from BC. It was documented that protein corona – external layer formed around nanostructures during their interactions with biological fluids (e.g., bovine serum present in cell culture medium) might reduce drug release (Mahmoudi, 2022; Li et al., 2021). It should also be noted that high crystallinity of BC’s may be another factor limiting drug release (Kenawy et al., 2002). Last but not least, pH is a parameter influencing release of active substances from drug carriers. Taokaew et al. revealed a significantly higher release rate of polyphenol and α-mangostin in PBS (pH 7.4) than in the acetate buffer solution (pH 5.5) (Taokaew et al., 2014). As tumour tissue characterizes more acidic extracellular pH (0.3–0.7 lower) than normal cells (Hao et al., 2018), so controlled liberation of anticancer compounds in the acidic environment is a highly desirable feature of localized drug delivery systems (Zhao et al., 2016; Zhuo et al., 2020). In the current study, pH of PBS and medium was constant (pH 7.4) and could not influence the release study.

*E. angustifolium* is a plant known for its anti-inflammatory, antibacterial, antioxidant, and antiproliferative properties, mostly related to high polyphenols content (Dreger et al., 2020). Therefore, in our study, the plant extract cumulative absorption and release profiles were determined based on the total polyphenol content. Due to the fact that those compounds produce a very broad range of biological effects, total content of polyphenols is often the first parameter illustrating biological value of plant material. Polyphenol composition of *E. angustifolium* includes mainly flavonoids, such as rutin, quercetin, and kaempferol, as well as phenolic acids, among others, chlorogenic acid, gallic acid, 4-hydroxybenzoic acid, 3,4-dihydroxybenzoic acid, caffeic acid (Zagórska-Dziok et al., 2021; Lasinskas et al., 2020) (Maruška et al., 2014; Ruszová et al., 2013). This abundant composition of *E. angustifolium* has been confirmed in our previous studies (Nowak et al., 2021c; Nowak et al., 2021b; Nowak et al., 2021a). However, the oral bioavailability of polyphenols is limited by food digestion and microbiota activity, which justifies application of gastro-resistant carriers that could preserve the bioactive components of *E. angustifolium*, enhancing their absorption (Dacrema et al., 2020). The use of carriers and local delivery of EAE will allow to overcome individual variability related to differences and composition of the gastrointestinal microorganisms (Piwowarski et al., 2016; Dabulici et al., 2020).

In the available literature, the anticancer effects of polyphenols are also well-documented (Asanarong et al., 2021; Niedzwiecki et al., 2016). Their potential activity as anticancer compounds is primarily attributed to their antioxidant properties, among others, strong radical scavenging, metal chelating, modifying endogenous defence mechanisms such as catalase (CAT), glutathione peroxidase (GPx), superoxide dismutase (SOD), enhancing glutathione (GSH) redox status, and regulating various proteins and transcription factors, such as nuclear factor erythroid 2-related factor 2 (Nrf2). Moreover, this specific group of compounds shows antiproliferative activity and prevention of migration and metastasis of neoplastic cells (Abotaleb et al., 2020; Kim et al., 2012). Strong antiproliferative activity of *E. angustifolium* extracts was observed against both normal and human prostate cancer cells, justifying its traditional use in urogenital diseases (Stolarczyk et al., 2013; Vitalone et al., 2001). Our study revealed that HT-29 cells presented the highest sensitivity to the tested extract with IC_50_ (61.73 ± 6.42 µM), three times lower than normal human cells, which may suggest the selectivity of *E. angustifolium* against some cancers. Kowalik et al. evaluated anticancer activity of digested aqueous extracts of fireweed, which inhibited proliferation of HT-29 cells and simultaneously stimulated cell division of the human colon epithelial cell line CCD 841 CoTr (Kowalik et al., 2022). At a concentration of 250 μg/mL, the cell proliferation varied from 27% (in the case of cancer) to 128% for normal cells. It should be pointed out that these differences in proliferation rate (measured by MTT assay) were observed only after 96 h (but not 24 h). Given the above data, it seems important to provide the extract in a form (e.g., immersed in BC) that enables its stable release over a longer time to ensure effective concentrations of active compounds at the site of action. Surprisingly, although the total amount of phenolic compounds in *E. angustifolium* infusion decreased after *in vitro* digestion (Kowalik et al., 2022; Dacrema et al., 2020), polyphenols such as quercetin or kaempferol, which are responsible for the cytotoxic effect of the extract on cancerous colon cells, remained at a sufficiently high level (Kowalik et al., 2022). Similarly, promising activity against colon cancer cells was seen with a YATROS, a herbal food supplement consisting of hydroalcoholic extracts of *Epilobium* (*E. parviflorum*, *E. angustifolium*, *E. hirsutum*) (40%), *Urtica dioica* (40%) and *Evernia prunastri* (20%). Moreover, synergistic inhibitory effects on cell growth, DNA synthesis, and cell cycle distribution were observed when YATROS was combined with 5-fluorouracil, contributing to a reduction in the effective dose and exposure time to drug against murine colon carcinoma C26 cell line (Zunino et al., 2017).

In our study, PrestoBlue assay results confirmed the biocompatibility of empty BC (with cell viability equal to nearly 100%) and dose and time-dependent cytotoxicity of the released EAE. In detail, plant extract released from BC-2.5%EAE significantly reduced the viability of HT-29 cells to 18.16% and 6.15% after 48 and 72 h of treatment, respectively. In the case of BC-1%EAE, prolonged incubation time was required to diminish cancer cell viability significantly. BC films containing the ethanolic extract of mangosteen peel were reported to produce significant cytotoxic effects against melanoma (B16) and breast cancer (MCF-7) cells. Subtaweesin et al. confirmed utility of BCs as the matrix for the slow local release of curcumin as a promising method to overcome the problems associated with its low oral bioavailability, instability at physiological pH, and rapid intestinal metabolism (Subtaweesin et al., 2018).

Similarly, Cacicedo et al. revealed that doxorubicin (DOX) loaded into BC-Alg films allowed to avoid drug crystallite formation and precipitation as well as contributed to a higher anticancer effect against HT-29 cells compared to the free drug (Cacicedo et al., 2016). In a further study, Caicedo et al. investigated a hybrid biomaterial consisting of BC, lipid nanocarriers, and DOX for local drug delivery into a mouse orthotopic breast cancer model (Cacicedo et al., 2018). The authors revealed that sustained release of the drug contributed to a significant reduction of tumour growth (more efficient than intratumorally administered free DOX), metastasis incidence, and local chemotherapeutic toxicities (e.g., inflammation, edema, and necrosis). Zhang et al. developed a more advanced BC-based tool for combined chemotherapy and photodynamic therapy, loaded with laser-sensitized magnetic nanoparticles, DOX, and hematoporphyrin (Zhang et al., 2019). This complex system bypassed the stratum corneum barrier, enhanced transdermal drug delivery, and inhibited breast cancer growth to 80.4%. Hence, due to its features and potential to modify the bioavailability of drugs, BC is currently extensively studied as a drug carrier.

The mechanism of anticancer activity of *E. angustifolium* is associated with induction of apoptosis. In our study, exposure of HT-29 cells to the EAE extracts released from BC significantly increased the percentage of apoptotic cancer cells in a dose and time-dependent manner. Stolarczyk et al. showed that aqueous extracts from *Epilobium* sp. (E*. angustifolium L., E. parviflorum Schreb.* and *E. hirsutum L.*) induce apoptosis in human hormone-dependent prostate cancer cells (LNCaP) *via* the mitochondrial pathway (Stolarczyk et al., 2013). However, a simultaneously high percentage of necrotic cells, especially at lower extract concentrations, were noted, which might suggest an additional cytotoxicity mechanism related to generation of free radicals. Pei et al. showed that Oenothein B (OeB), one of the most important active compounds found in *E. angustifolium*, effectively induced apoptosis *via* arrested cells in G1 and ROS-mediated PI3K/Akt/NF-κB signaling pathway in human non-small lung cancer cells (A549) (Pei et al., 2019). It should be emphasized that the biological activity of *E. angustifolium* may differ depending on the extraction method, parts of the plants used, and tested cells (Dreger et al., 2020; Kiss et al., 2006).

## 5 Conclusion

In the current study, the anticancer properties of ethanolic extract of *E. angustifolium* were confirmed. Moreover, we revealed that colon cancer cells (line HT-29) showed the highest sensitivity to the tested plant extract, which was demonstrated by the IC_50_ values, which were three times lower compared to normal human fibroblasts. Systemic adjuvant therapy is recommended in many cases of colon cancer to prevent tumor recurrence after surgery, and it is associated with a number of adverse effects. Therefore, new treatment strategies that deliver anticancer compounds directly to the target structures are urgently needed. In the current work, BC membranes were applied as a matrix for localized delivery of the plant extract with potential anticancer activity. In addition, a high initial dose of the extract and its satisfactory kinetic release profile would allow for avoiding limitations associated with its low oral bioavailability. Our study confirmed the biocompatibility of empty BC and the dose and time-dependent cytotoxicity of the BC-EAE associated with sustained polyphenols release. In conclusion, we have shown that BC membranes could be used as a carrier for localized and sustained delivery of plant extracts. However, the favorable physicochemical properties of BC, which enable effective loading and stable release of carried compounds, ensuring adequate concentrations at target sites over a long time, suggest its future potential application as carriers of anticancer drugs. Moreover, simplicity of BCs synthesis and a wide spectrum of biological properties of *E. angustifolium* may indicate wider and more common use of membranes in transdermal therapy. Future studies will be essential in verification of this strategy in clinical settings.

## Data Availability

The original contributions presented in the study are included in the article/Supplementary Material, further inquiries can be directed to the corresponding author.
