# Supplementary material for: Anticancer properties of bacterial cellulose membrane containing ethanolic extract of Epilobium angustifolium L
Source: Front Bioeng Biotechnol. 2023 Feb 20;11:1133345. doi: 10.3389/fbioe.2023.1133345 (PMC9986418; doi:10.3389/fbioe.2023.1133345)
Supplement: Supplementary file 1 [file DataSheet1.docx]

Supplementary Material

Anticancer properties of bacterial cellulose membrane containing ethanolic extract of *Epilobium angustifolium L.*

**Magdalena Perużyńska ^1*^, Anna Nowak ^2^, Radosław Birger ^1^, Paula Ossowicz-Rupniewska ^3^, Maciej Konopacki ^4^, Rafał Rakoczy ^4^, Łukasz Kucharski ^2^, Karolina Wenelska ^5^, Adam Klimowicz ^2^, Marek Droździk ^1^, Mateusz Kurzawski ^1^**

^1^ Pomeranian Medical University in Szczecin, Department of Experimental and Clinical Pharmacology, Powstańców Wielkopolskich Avenue 72, 70-111 Szczecin, Poland

^2^ Pomeranian Medical University in Szczecin, Department of Cosmetic and Pharmaceutical Chemistry, Powstańców Wielkopolskich Avenue 72, 70-111 Szczecin, Poland

^3^ West Pomeranian University of Technology in Szczecin, Faculty of Chemical Technology and Engineering, Department of Chemical Organic Technology and Polymeric Materials, Piastów Avenue 42, 71-065 Szczecin, Poland

^4^ West Pomeranian University of Technology in Szczecin, Faculty of Chemical Technology and Engineering, Department of Chemical and Process Engineering, Piastów Avenue 42, 71-065 Szczecin, Poland

^5^ West Pomeranian University of Technology in Szczecin, Faculty of Chemical Technology and Engineering, Department of Nanomaterials Physicochemistry, Piastów Avenue 45, 70-311 Szczecin, Poland

***Correspondence:** Magdalena Perużyńska magdalena.peruzynska@pum.edu.pl


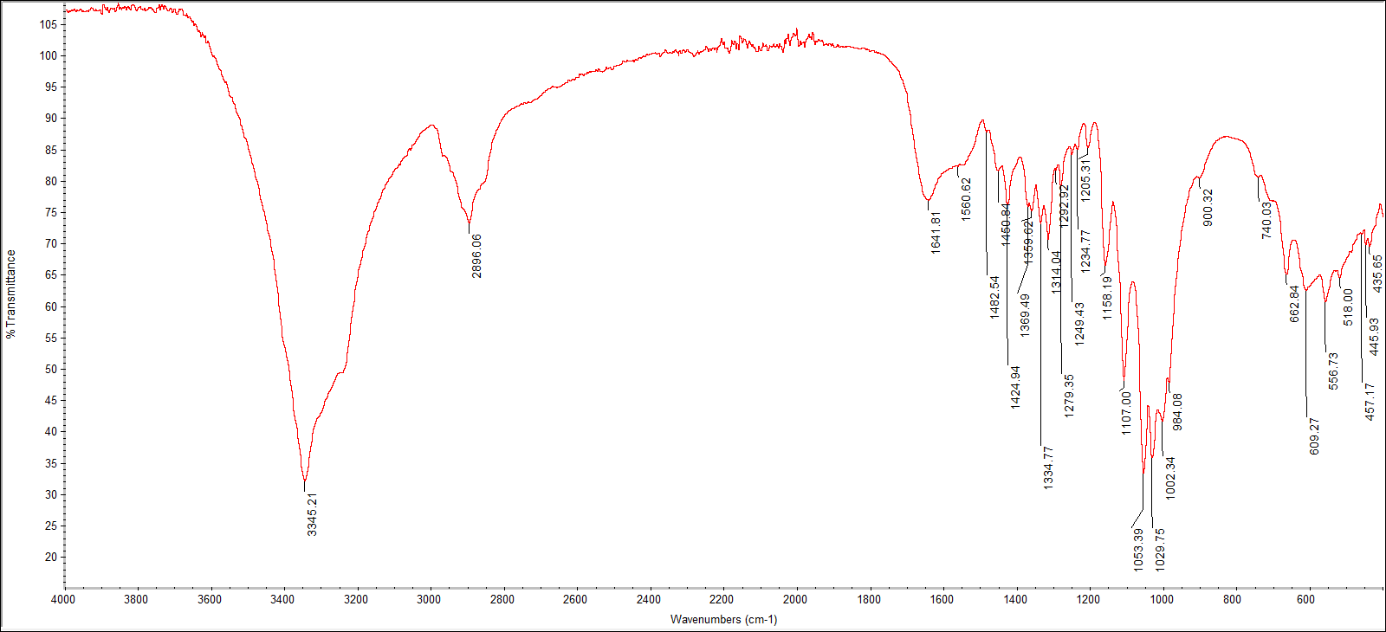


**Figure S1.** FTIR-ATR spectrum of empty BC.


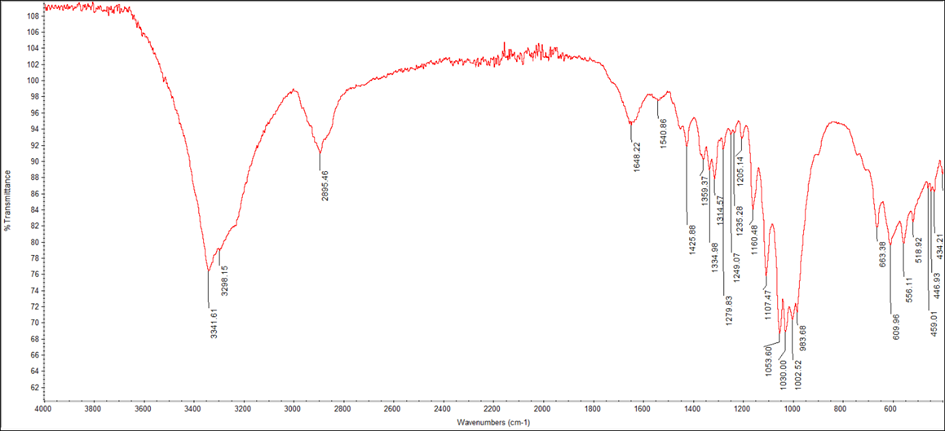


**Figure S2.** FTIR-ATR spectrum of BC-0.25%EAE.


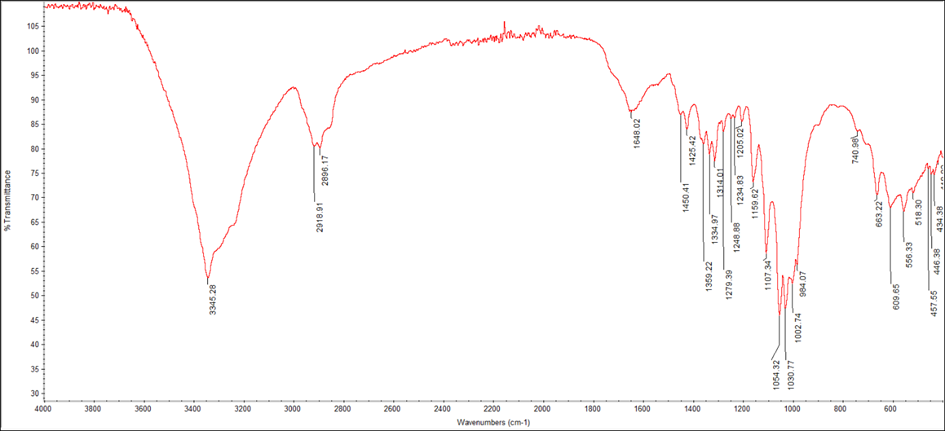


**Figure S3.** FTIR-ATR spectrum of BC-0.5%EAE.


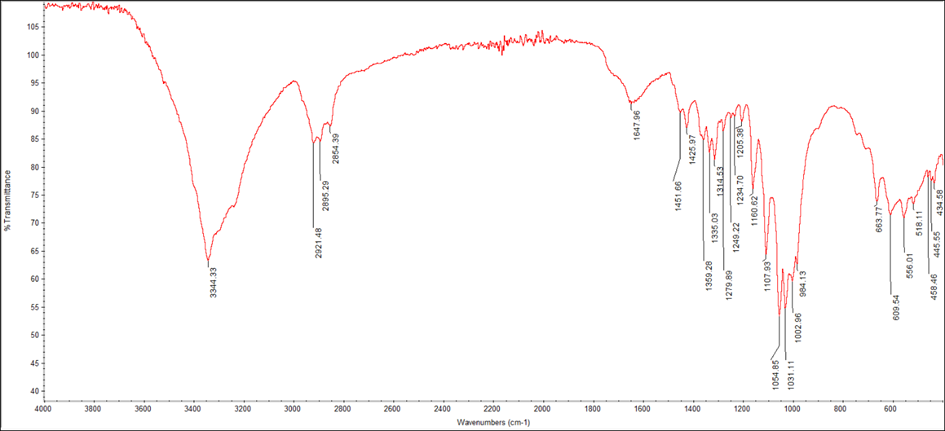


**Figure S4.** FTIR-ATR spectrum of BC-1%EAE.


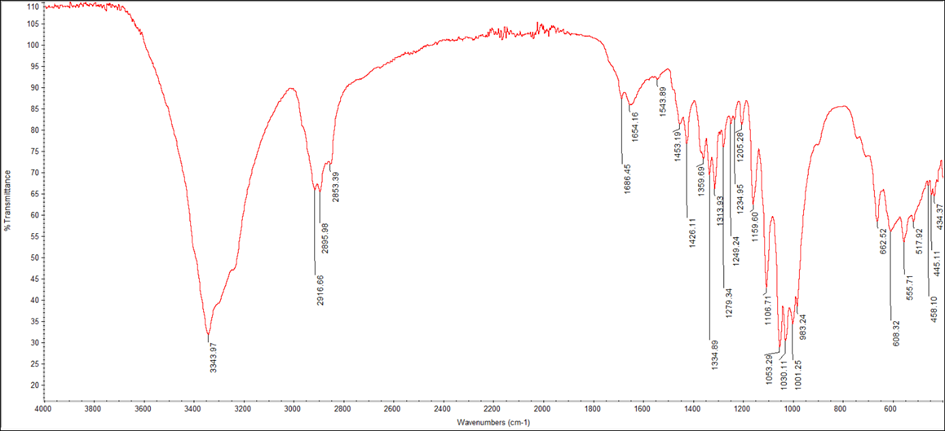


**Figure S5.** FTIR-ATR spectrum of BC-2.5%EAE.


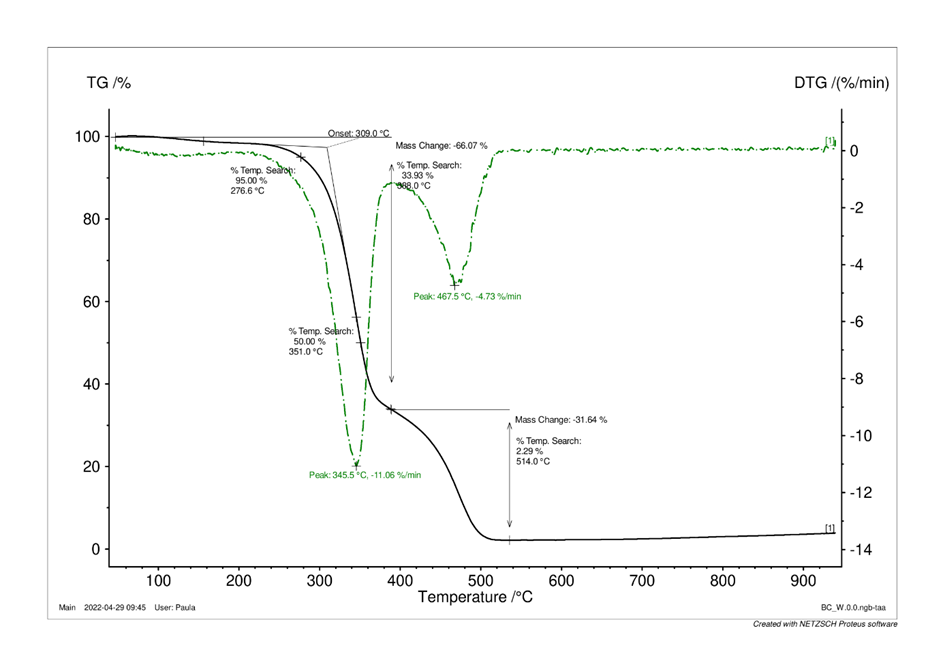


**Figure S6.** The TG and DTG curves of empty BC.


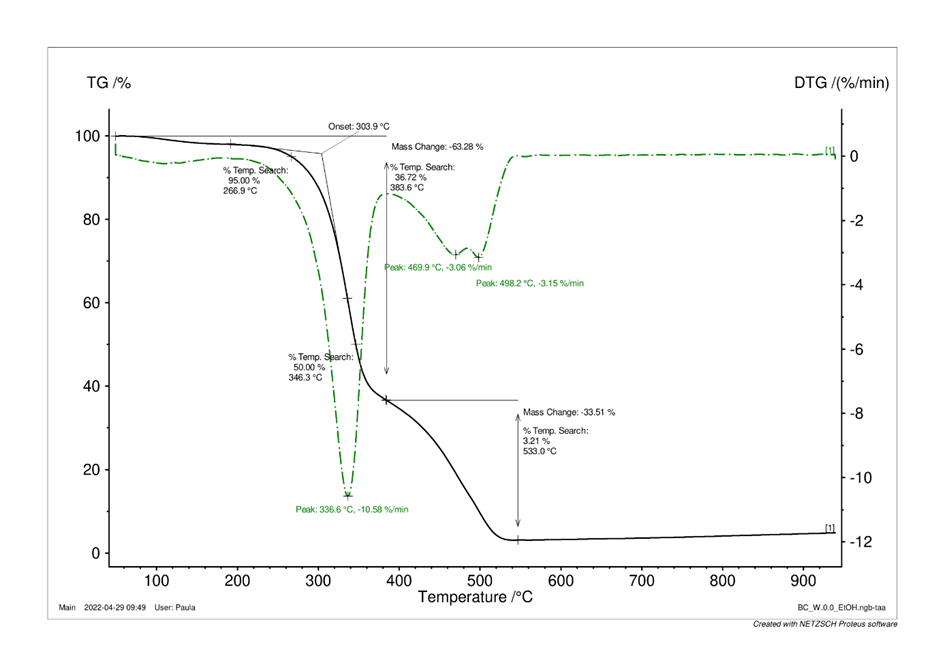


**Figure S7.** The TG and DTG curves of BC-EtOH.


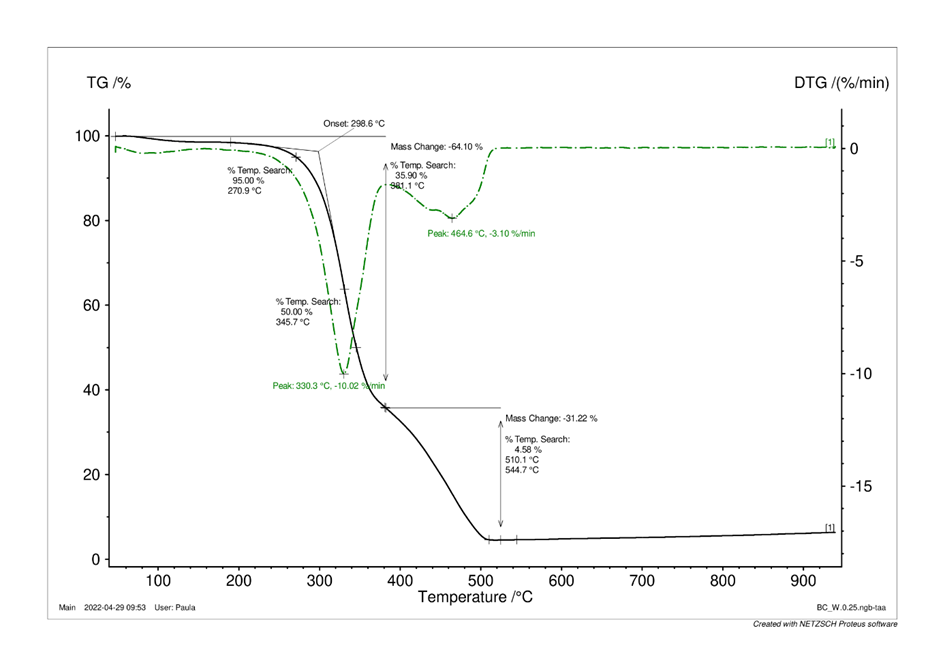


**Figure S8.** The TG and DTG curves of BC-0.25%EAE.


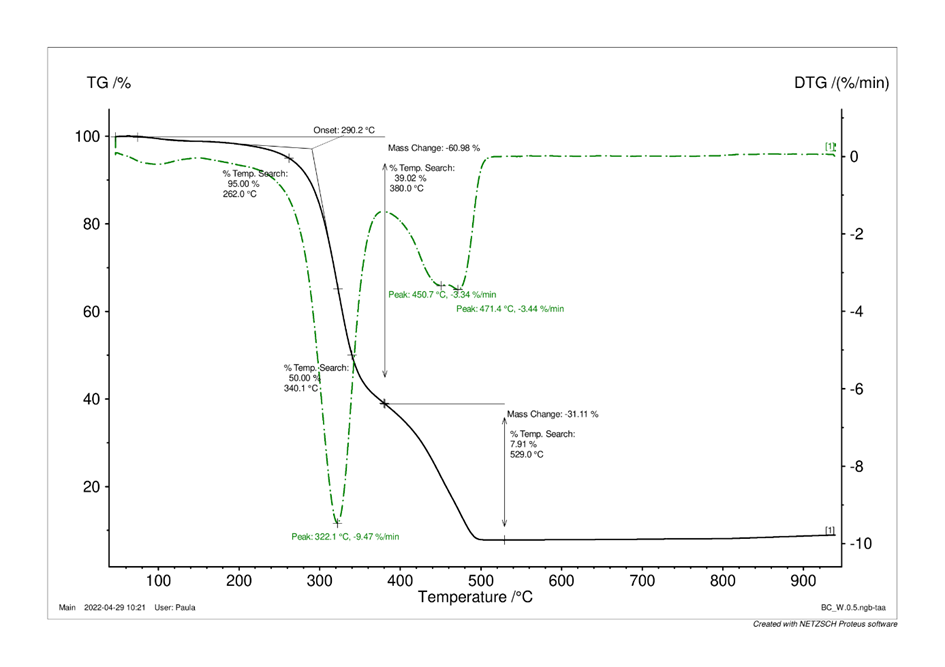


**Figure S9.** The TG and DTG curves of BC-0.5%EAE.


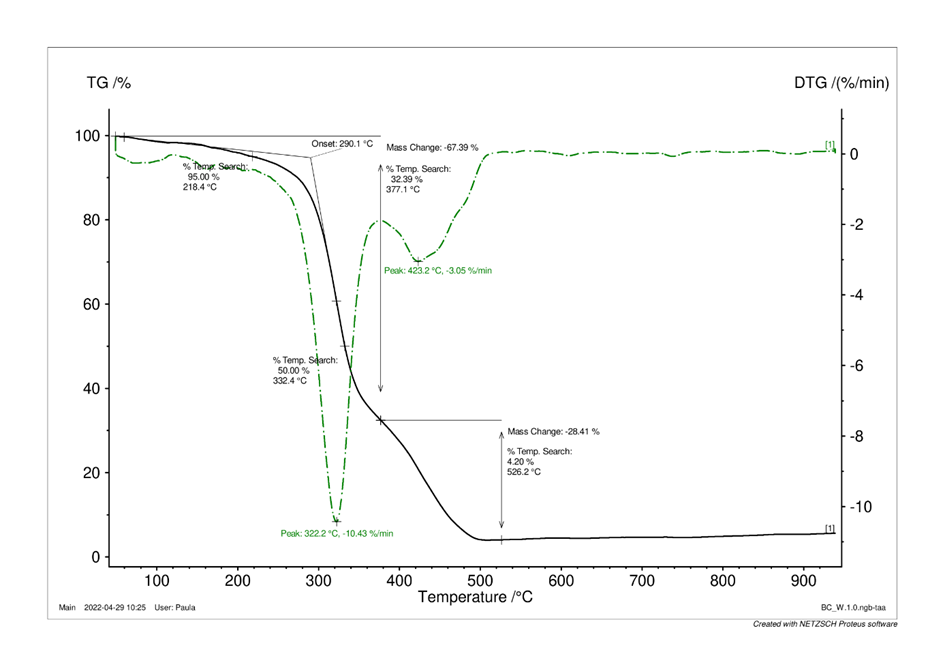


**Figure S10.** The TG and DTG curves of BC-1%EAE.


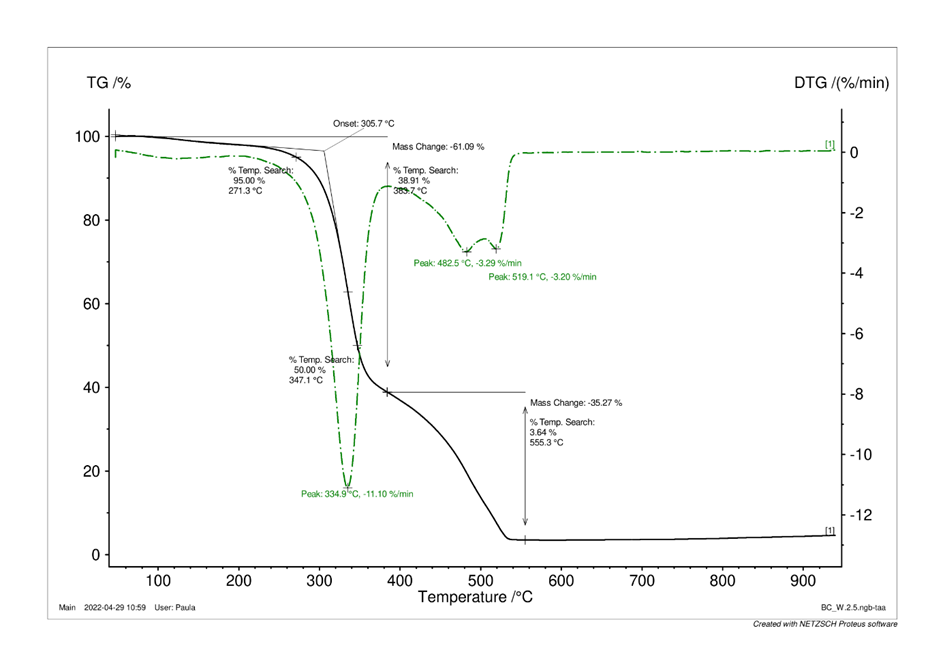


**Figure S11.** The TG and DTG curves of BC-2.5%EAE.


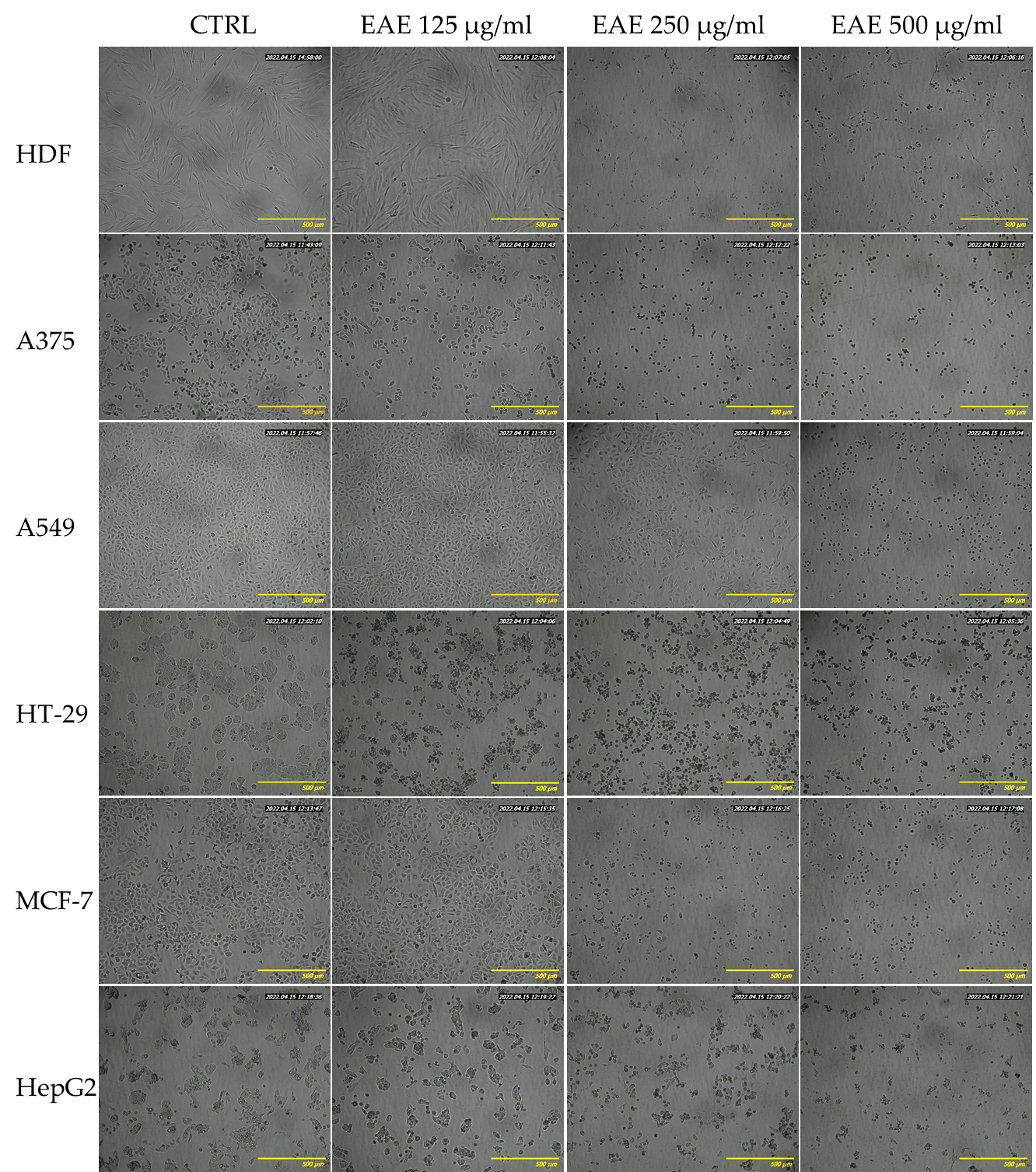


**Figure S12**. Optical microscopy images of tested cells after 48 h incubation with the highest concentrations of EAE, where the differences between control and treaded cells were the most visible.
